# Meeting Report: BioSharing at ISMB 2010

**DOI:** 10.4056/sigs/1403501

**Published:** 2010-12-04

**Authors:** Dawn Field, Susanna Sansone, Edward F. DeLong, Peter Sterk, Iddo Friedberg, Pascale Gaudet, Susanna Lewis, Renzo Kottmann, Lynette Hirschman, George Garrity, Guy Cochrane, John Wooley, Folker Meyer, Sarah Hunter, Owen White, Brian Bramlett, Susan Gregurick, Hilmar Lapp, Sandra Orchard, Philippe Rocca-Serra, Alan Ruttenberg, Nigam Shah, Chris Taylor, Anne Thessen

**Affiliations:** 1 Centre for Ecology & Hydrology, Maclean Building, Benson Lane, Crowmarsh Gifford, Wallingford, Oxfordshire, OX10 8BB, UK; 2 University of Oxford, Oxford e-Research Centre, Oxford, UK; 3 Department of Biological Engineering, Massachusetts Institue of Technology, Cambridge, MA 02138, USA; 4 Department of Microbiology, Miami University, Oxford OH 45056, USA; 5 Department of Computer Science and Software Engineering, Miami University, Oxford OH 45056, USA; 6 Swiss Institute of Bioinformatics, CMU - 1, rue Michel Servet, CH-1211 Geneva 4, Switzerland; 7 Lawrence Berkeley National Laboratory, One Cyclotron Road, Berkeley, CA, USA; 8 Microbial Genomics Group, Max Planck Institute for Marine Microbiology & Jacobs University Bremen, D-28359 Bremen, Germany; 9 Information Technology Center, The MITRE Corporation, 202 Burlington Road, Bedford, MA 01730, USA; 10 Department of Microbiology and Molecular Genetics, Michigan State University, East Lansing, Michigan 48824, USA; 11 European Molecular Biology Laboratory (EMBL) Outstation, European Bioinformatics Institute (EBI), Wellcome Trust Genome Campus, Hinxton, Cambridge CB10 1SD, UK; 12 University of California San Diego, 9500 Gilman Drive, La Jolla, CA 92093, USA; 13 Argonne National Laboratory, 9700 South Cass Avenue, Argonne, IL 60439, USA; 14 Institute for Genome Sciences, University of Maryland School of Medicine, Baltimore, MD 21201, USA; 15 Lux Bio Group, 121 SW Morrison Street, Suite 1550, Portland, Oregon 97204, USA; 16 Department of Energy, Biological Systems Science Division, 1000 Independence Ave., Washington, DC 20585, USA; 17 National Evolutionary Synthesis Center (NESCent), Durham, NC 27705, USA; 18 Science Commons, c/o Massachusetts Institute of Technology Computer Science and Artificial Intelligence Laboratory, Building 32-386D, 32 Vassar Street, Cambridge, MA 02139, USA; 19 Stanford Center for Biomedical Informatics Research, Medical School Office Building X-215, 251 Campus Drive, Stanford, CA 94305, USA; 20 Marine Biological Laboratory, 7 MBL Street, Woods Hole, MA 02543, USA

## Abstract

This report summarizes the proceedings of the one day BioSharing meeting held at the Intelligent Systems for Molecular Biology (ISMB) 2010 conference in Boston, MA, USA This inaugural BioSharing event was hosted by the Genomic Standards Consortium as part of its *M3 & BioSharing special interest group (SIG)* workshop. The BioSharing event included invited talks from a range of community leaders and a panel discussion at the end of the day. The panel session led to the formal agreement among community leaders to join together to promote cross-community knowledge exchange and collaborations. A key focus of the newly formed Biosharing community will be linking up resources to promote real-world data sharing (virtuous cycle of data) and supporting compliance with data policies through the creation of a one-stop-portal of information. Further information about the newly established BioSharing effort can be found at http://biosharing.org.

## Introduction

The M3 & Biosharing special interest group (SIG) hosted by the Genomic Standards Consortium (GSC, [1]) at the Intelligent Systems in Molecular Biology (ISMB) 2010 conference explored the latest concepts, informatics resources, and standards that are being developed to cope with the analysis of vast quantities of metagenomic data [2]. As part of the outreach of the GSC to other data-sharing communities, the second day of the SIG served as the inaugural meeting of the BioSharing initiative [3]. During this day-long meeting of communities interested in data-sharing, the focus shifted to addressing the wider issue of how to increase engagement between funding agencies and researchers to build better data policies to promote real-world data sharing through the use of standards.

### An increased focus on ‘omics data sharing

Data sharing policies are emerging in response to increased funding for high-throughput approaches in major bioscience domains [4], including genomics and functional genomics. But despite their commonalities, the policies are heterogeneous by nature, given the different types of communities served and the data types they cover. In parallel, an escalating number of community-developed standards (minimal requirements checklists [5], ontologies [6], and file-formats) operate to support the harmonization of the reporting process, so that different experiments can be compared or integrated. The proliferation of these standardization efforts is a positive sign of community engagement, but it also brings with it new sociological and technological challenges - creating interoperability and avoiding unnecessary overlap and duplication of effort that hampers their wider uptake.

The BioSharing initiative [3] seeks to facilitate a broader dialogue among funders, journals, standards developers, technology developers and researchers on the critical issue of data sharing within the metagenomics community and beyond. To help encourage this dialogue, 14 community leaders were invited to come together to present overviews of their community-level efforts and discussion how to move forward. This report briefly summarizes the presentations and discussions of this BioSharing day.

## BioSharing - Towards real-world data sharing

The agenda of the day was designed to focus on the intersections of science, standards, and policy. Dawn Field and Susanna Sansone, founding members of BioSharing, described how the concept of a BioSharing community stemmed from their recent article *Omics data sharing*, written in collaboration with a large number of funders developing and maintaining data sharing policies [4]. The purpose of this BioSharing day was to bring together, for the first time, representatives of a variety of these communities to kick-start cross-community interactions and achieve agreement on how to move forward.

### The BioSharing Plenary Talk - Strong Data Policies from Funding Agencies

The day opened with a plenary talk from **Susan Gregurick**, a co-author of the ‘Omics data sharing paper [4] and representative of the **Department of Energy** (DOE [7]), which maintains a strong data sharing policy within its Genomes to Life (GTL) program. Dr. Gregurick gave an overview of the mission of the DOE and its strong commitment to data sharing. To help set the stage for the discussion at this meeting, she also announced that the National Science Foundation (NSF,[8]) would be implementing a new approach to data stewardship through the ‘Data Management Plan’ requirement in future grants. This approach will likely be rolled out to other federal funding agencies. This will require researchers to be increasingly familiar with existing and planned data sharing solutions for their particular area of research.

### Community Introductions

All remaining presentations of this day were dedicated to community introductions by community leaders. In turn, each community representative was asked to state the purpose and current status of work in their community, its mission, and to highlight specific activities it might be undertaking to work at the interface of the many activities covered within BioSharing, such as ontologies, checklists, data formats, enabling technologies, scientific publications, databases, and data policies. A total of 12 formalized community-level projects were described covering the perspectives of checklists, ontologies, software, databases, journals and collaborative data sharing efforts (Table 1). In addition, there were talks to represent the general ‘database’ and ‘natural language processing (BioNLP) communities from Guy Cochrane (EBI) and Lynette Hirschman (MITRE). Combined, this group covered a wide range of expertise and projects. The need to ‘close the virtuous cycle’, through increased collaboration at the intersections of these communities, was a common theme. All presentations are available online from the BioSharing website.

**Table 1:** List of communities, their missions, and community representative, in the order in which they were presented at the first BioSharing workshop.

**Community**	**Scope and mission**	**Presenting community representative**
Genomic Standards Consortium http://gensc.org/	**standards** for describing genomes, metagenomes and gene marker sequences	Dawn Field (NERC Centre for Ecology and Hydrology) [9]
		
The HUPO Proteomics Standards Intiative (PSI) http://www.psidev.info/	standards for data representation in **proteomics** to facilitate data comparison, exchange and verification	Sandra Orchard (European Bioinformatics Institute) [10]
		
MIBBI http://mibbi.org/	brings together over 30 **checklist** communities	Chris Taylor (European Bioinformatics Institute) [5]
		
The OBO Foundry http://www.obofoundry.org/	goal of creating a suite of orthogonal, interoperable reference **ontologies**	Suzi Lewis (Lawrence Berkeley National Laboratory) [6]
		
International Society for Biocuration (ISB) http://www.biocurator.org	promoting the work of **biocurators** both to users of the curated biological databases and to the funding agencies.	Pascale Gaudet (Northwestern University/Swiss Institute of Bioinformatics) [11]
		
The Data Conservancy http://www.dataconservancy.org	promote **data sharing** and reuse across the spectrum of biology in alignment with the NSF	Anne Thessen (Woods Hole)
		
The Standards in Genomic Sciences journal http://standardsingenomics.org/	an open-access, standards-supportive **journal** for rapid dissemination of range of article types	George Garrity (Michigan State University) [12]
		
Science Commons http://sciencecommons.org/	design strategies and tools for faster, more efficient **web-enabled scientific research**.	Alan Ruttenberg (Science Commons)
		
The ISA Infrastructure http://www.isa-tools.org	open source **software suite** for data sharing of experimental metadata	Philippe Rocca-Serra (University of Oxford) [13]
		
The Dryad Digital Repository: Published data as part of the greater data ecosystem http://datadryad.org/	digital **data repository** for basic and applied biosciences	Hilmar Lapp (NESCent) [4]
		
National Center for Biomedical Ontology (NCBO) http://www.bioontology.org/	create software and **Web services** for the application of principled ontologies in biomedical science	Nigam Shah (Stanford University)
		
Digital Biology Foundation: open software community in life sciences http://digibio.org/	**foundation** to support for the intersection of life sciences and software development	Brian Bramlett, (Lux Bio Group)

## Panel Discussion: developing a vision for the future

Chaired by Dawn Field and Susanna Sansone, the Panel discussion at the end of the day included all speakers. This was the first time many of these community leaders had met in person and all agreed on the importance of this meeting as the first step in working together. All agreed to move forward as a group to build linkages through a BioSharing effort. There was strong interest in a follow up meeting. To support further activities, it was agreed that the BioSharing forum will have a combination of targeted and open-attendance meetings, normally as part of larger meetings so as to reach as broad an audience as possible, especially potential grant awardees and therefore future users of standards. The forum will utilize all possible means to disseminate information (such as RSS feeds, position papers, presentations). Following this successful meeting, a statement of purpose was formulated. It can be found in full on the BioSharing website [3]. Also, as a result of the meeting Pascale Gaudet took forward the development of a minimum information checklist for describing databases, BioDBCore as a BioSharing project led by her community, the International Society of Biocuration (ISB) [14].

### BioSharing Forum Statement of Purpose

The BioSharing community will work at the global level to build stable linkages between funders, implementing data sharing policies, and well-constituted standardization efforts in the biosciences domain, to expedite the communication and the production of an integrated standards-based framework for the capture and sharing of high-throughput genomics and functional genomic bioscience data.

**This overall objective has several components, each of which can be further decomposed:**
- Web site to centralize bioscience data policies, reporting standards and links to other related portals
  o Providing a “one-stop shop” for those seeking data sharing policy documents and information about the standards and technologies that support them.
  o Exposing core information on well-constituted, community-driven standardization efforts and link to their reporting standards (checklists, ontologies and file-formats), documentation, training material, news and contact point.
  o Linking to existing portals or new resources (to be developed collaboratively with other groups and initiatives) for those seeking information on systems serving or implementing the standards.
- Communication forum for funders and leaders of the standardization efforts to achieve harmonization and mutual support
  o Lobbying for intra-harmonization within these two groups to promote:
    - exchange of ideas and policy components among public and private funders, and between funders and finding recipients, to ensure that the difference among the policies (such as the reporting standards that may be supported) ultimately do not impede seamless interoperability of the data.
    - collaboration among the standardization efforts to create interoperable reporting standards and to avoid unnecessary overlap, duplication of effort and incompatible tools.
  o Identifying a mutual support system between the two stakeholder groups to ensure:
    - funding agencies are abreast with challenges the standardization efforts face and can provide targeted funds to sustain their development and maintenance;
    - when community-developed standards are mature and appropriate standards-compliant systems become available these are channeled to the appropriate funding agencies, which in turn endorse them in agency data sharing policies, thus achieving wider harmonization of the data.
